# Polyphosphate in *Lactobacillus* and Its Link to Stress Tolerance and Probiotic Properties

**DOI:** 10.3389/fmicb.2018.01944

**Published:** 2018-09-07

**Authors:** Cristina Alcántara, José M. Coll-Marqués, Carlos Jadán-Piedra, Dinoraz Vélez, Vicenta Devesa, Manuel Zúñiga, Vicente Monedero

**Affiliations:** ^1^Laboratory of Lactic Acid Bacteria and Probiotics, Instituto de Agroquímica y Tecnología de Alimentos, Consejo Superior de Investigaciones Científicas, Valencia, Spain; ^2^Trace Elements Group, Instituto de Agroquímica y Tecnología de Alimentos, Consejo Superior de Investigaciones Científicas, Valencia, Spain

**Keywords:** *Lactobacillus*, probiotic, mercury, polyphosphate, polyphosphate kinase

## Abstract

The synthesis of the inorganic polymer polyphosphate (poly-P) in bacteria has been linked to stress survival and to the capacity of some strains to sequester heavy metals. In addition, synthesis of poly-P by certain strains of probiotic lactobacilli has been evidenced as a probiotic mechanism due to the homeostatic properties of this compound at the intestinal epithelium. We analyzed the link between poly-P synthesis, stress response, and mercury toxicity/accumulation by comparing wild-type strains of *Lactobacillus* and their corresponding mutants devoid of poly-P synthesis capacity (defective in the poly-P kinase, *ppk*, gene). Results showed that resistance to salt (NaCl) and acidic (pH 4) stresses upon *ppk* mutation was affected in *Lactobacillus casei*, while no effect was observed in two different *Lactobacillus plantarum* strains. Inorganic [Hg(II)] and organic (CH_3_Hg) mercury toxicity was generally increased upon *ppk* mutation, but no influence was seen on the capacity to retain both mercurial forms by the bacteria. Notwithstanding, the culture supernatants of *ppk*-defective *L. plantarum* strains possessed a diminished capacity to induce HSP27 expression, a marker for cell protection, in cultured Caco-2 cells compared to wild-type strains. In summary, our results illustrate that the role of poly-P in stress tolerance can vary between strains and they reinforce the idea of probiotic-derived poly-P as a molecule that modulates host-signaling pathways. They also question the relevance of this polymer to the capacity to retain mercury of probiotics.

## Introduction

Polyphosphate (poly-P) is an inorganic phosphate polymer of hundreds of phosphates that can be synthesized in bacteria by the action of the enzyme poly-P kinase (PPK) (Rao et al., 2009). Many bacterial strains possess the capacity to intracellularly accumulate this compound in high amounts in the form of poly-P granules and this characteristic is relevant, for example, in the process of biological phosphate removal in wastewater treatment plants (Yuan et al., 2012). Poly-P has diverse physiological roles, some of them not totally understood yet. Besides its function as a storage compound, poly-P can be involved in stress response [e.g., starvation, acid or oxidative stresses (Gray and Jakob, 2015), virulence, motility or biofilm formation, among others physiological processes (Albi and Serrano, 2016)].

References to the physiological roles of poly-P in the food- and health-related genus *Lactobacillus* are scarce and they were until recently related to the capacity of *Lactobacillus plantarum* to accumulate Mn^2+^ complexed to poly-P, in a mechanism of oxidative stress defense (Archibald and Fridovich, 1982). In addition to this, very few works described the presence of poly-P accumulation in this genus (Aprea et al., 2005). However, a survey of *Lactobacillus* genomes revealed that the presence of genes encoding PPK enzymes (*ppk*) is common in lactobacilli (Alcantara et al., 2014). Furthermore, the *ppk* gene was linked to the occurrence of poly-P granules, which could be massively accumulated in some strains (Alcantara et al., 2014). The physiological roles of poly-P in lactobacilli have not been completely identified. Besides the aforementioned role in oxidative stress resistance in *L. plantarum*, intracellular poly-P accumulation has been linked to resistance to several stresses in *Lactobacillus casei* BL23 (Alcantara et al., 2014) and in *Lactobacillus rhamnosus* CRL1505 (Correa Deza et al., 2017).

Due to its elevated negative charge density, it has been suggested that poly-P could aid in the bioremediation of toxic trace elements, acting as a chelating agent for positively charged metals such as mercury or cadmium. Thus, transgenic plants that produce poly-P via the expression of bacterial PPKs have been constructed that showed an increased capacity for mercury accumulation (Nagata et al., 2010; Chang et al., 2015). Similarly, recombinant *E. coli* strains with increased levels of poly-P as a result of PPK overexpression also decreased their sensitivity toward mercury and displayed an increased inorganic mercury accumulation (Pan-Hou et al., 2002; Ruiz et al., 2011).

Many *Lactobacillus* strains have a great capacity to sequester heavy metals. This observation has led to explore their use as tools to reduce the bioaccessibility of metals in the gastrointestinal tract and some strains have been tested in animal and human trials (Bisanz et al., 2014; Zhai et al., 2014; Ojekunle et al., 2017). However, the relevance of poly-P synthesis in this process has not been studied. Poly-P has also been identified as the molecule produced in the supernatants of the probiotic *Lactobacillus brevis* SBC8803 strain that helped to the maintenance of intestinal homeostasis in models of intestinal injury (Segawa et al., 2011). Thus, poly-P participates in the induction of cytoprotective heat-shock proteins through the regulation of the p38 MAPK pathway via its binding to integrin β1 (Segawa et al., 2011). Further studies on the role of this probiotic-derived poly-P proved that it suppressed inflammation in dextran sulfate sodium (DSS)- and 2,4,6-trinitrobenzenesulfonic acid (TNBS)-induced colitis in mice (Kashima et al., 2015) and enhanced intestinal barrier function (Tanaka et al., 2015).

In this study, we employed *Lactobacillus* strains mutated in their *ppk* genes to test the relevance of synthesized poly-P for stress tolerance, mercury complexation by the bacteria and for the induction of cytoprotective HSP27 in cultured cells. We showed that while *ppk* mutants confirmed the effect of poly-P in stress response and its functionality as a probiotic factor, mercury complexation was not related to poly-P synthesis under our experimental conditions.

## Materials and Methods

### Strains and Culture Conditions

Bacterial strains used in this study are listed in **Table 1**. Lactobacilli were routinely grown in MRS medium (Difco) at 37°C under static conditions. When required, MEI medium (Landete et al., 2010) without cysteine (0.5% yeast extract, 0.5% tryptone, 0.4% K_2_HPO_4_, 0.5% KH_2_PO_4_, 0.02% MgSO_4_⋅7H_2_O, 0.005% MnSO_4_, 1 ml of Tween 80 per liter and 0.5% glucose) was used. *Escherichia coli* was used as a cloning host and it was grown in Luria-Bertani broth at 37°C under agitation. Erythromycin and chloramphenicol used to select *Lactobacillus* transformants were added at 5 μg/ml, while ampicillin was added at 100 μg/ml for *E. coli*. When required, agar plates were prepared by adding 1.8% (w/v) agar.

**Table 1:** Strains and plasmids used in this study.

Strain/plasmid	Characteristics	Origin or reference
***L. casei***		
BL23	Laboratory strain; wild type	Maze et al., 2010
BL379	BL23 *ppk*::pRV300; Ery^r^	Alcantara et al., 2014
***L. plantarum***		
WCFS1	Intestinal isolate	Kleerebezem et al., 2003
BL398	WCFS1 *ppk*::pRV300; Ery^r^	This work
Lpp+	Laboratory strain; wild type	This work
Lpp+ pVE6007	Lpp+ carrying pVE6007; *repA^TS^*, Cm^r^	This work
Lpp+ pVE6007 pORIppk	Lpp+ carrying pVE6007 and pORIppk; *repA*^TS^, Cm^r^, Ery^r^	This work
Lppk	Lpp+ *ppk*::pORI19; Ery^r^	This work
***E. coli***		
DH10B	F^−^ *mcrA* Δ(*mrr-hsdRMS-mcrBC*) Φ80d*lacZ*ΔM15 Δ*lac*X74 *endA1 recA1 deoR* Δ(*ara, leu*)7697 *araD*139 *galU galK nupG rpsL*	Grant et al., 1990
EC101	[F’ *traD36 proA^+^B^+^ lacI^q^ lacZ*ΔM15] Δ*(lac-proAB*) *supE thi repA*^+^(pWV01). Host for propagating pORI19-derivatives	Law et al., 1995
**Plasmid**		
pRV300	Integrative plasmid; *lacZ’*, Amp^r^, Ery^r^	Leloup et al., 1997
pORI19	Integrative plasmid lacking *repA* gene; *LacZ’*, Ery^r^	Law et al., 1995
pVE6007	Thermosensitive *repA* from pWV01 (*repA^TS^*); Cm^r^	Maguin et al., 1992
pRVppkW	pRV300 with a 504-bp internal fragment of WCFS1 *ppk* gene cloned at XhoI and EcoRI	This work
pORIppk	pORI19 with a 504-bp internal fragment of Lpp+ *ppk* gene cloned at PstI and EcoRI	This work

*Amp^r^, ampicillin resistance; Ery^r^, erythromycin resistance; Cm^r^, chloramphenicol resistance.*

### Mutant Construction

Chromosomal DNA from *Lactobacillus* was isolated from bacteria grown in 10 ml cultures with the DNA Isolation Kit for Cells and Tissues (Roche Applied Science). An internal 504-bp portion of the *L. plantarum* WCFS1 *ppk* gene (LP_0842) was amplified by PCR with the oligonucleotide pair ppkWF (5′-TTTTCTCGAGGGACTTATTAAAGGAAGTTC) and ppkWR (5′-TTTTGAATTCTTATTTTCTTCATCAAAACG). Restriction sites introduced for cloning are underlined. The obtained product was isolated with the Illustra GFX PCR DNA and Gel Band Purification Kit (GE Healthcare), digested with XhoI and EcoRI and ligated to the integrative vector pRV300 (Leloup et al., 1997) digested with the same enzymes, giving pRVppkW. A similar *ppk* fragment from the *L. plantarum* Lpp+ strain was amplified with the oligonucleotide pairs ppKFPst (5′-TTTTCTGCAGGGACTTATTAAAGGAAGTTC) and ppkWR and cloned into pORI19 (Law et al., 1995) digested with PstI and EcoRI, giving pORIppk. Both integrative plasmids were isolated from *E. coli* with the Illustra plasmidPrep Mini Spin Kit (GE Healthcare) and used to transform *L. plantarum* WCFS1 and *L. plantarum* Lpp+, respectively, by electroporation with a Gene-Pulser apparatus (Bio-Rad). *L. plantarum* competent cells were prepared as described by Aukrust and Blom (1992) with modifications. The strains were grown in 50 ml of MRS supplemented with 1% (w/v) glycine to an OD_550_ of 0.6. Cells were washed at 4°C with 1 volume of cold 1 mM MgCl_2_ followed by a wash with half volume of cold 30% polyethylene glycol (PEG) 1500, before being suspended in 500 μl of 30% PEG 1500. Forty microliters of this suspension were electroporated in 0.2 cm-path cuvettes at 1.5 kV, 25 μF and 400 Ω, the cell suspension was immediately dispersed in 1 ml of MRS and incubated for 2 h at 30°C before plating. *L. plantarum* WCFS1 transformants were selected in MRS plates containing 5 μg/ml erythromycin and the occurrence of the correct integration was checked by PCR with DNA isolated from the integrants and appropriate oligonucleotides hybridizing in the vector and outside the cloned *ppk* fragment. For *ppk* disruption in Lpp+, the strain was first transformed with plasmid pVE6007 (Maguin et al., 1992) and transformants were selected at 30°C with 5 μg/ml chloramphenicol. This allowed the subsequent transformation with pORIppk as pVE6007 provided the RepA protein required for the replication of pORIppk. One transformant carrying pVE6007 and pORIppk was propagated at 30°C and used to inoculate 5 ml of MRS at the non-permissive temperature (37°C) in the absence of antibiotics. Bacteria from this culture were plated on MRS plates plus 5 μg/ml of erythromycin and incubated at 37°C. Erythromycin-resistant clones were checked for chloramphenicol-negative phenotype at 30°C, which indicated loss of thermosensitive pVE6007 and chromosomal integration of the pORIppk plasmid. The presence of this integration at the *ppk* locus in selected clones was checked by PCR with appropriate oligonucleotides.

### Assays of Sensitivity to Low pH and High Salt Concentration

The growth of *L. casei*, *L. plantarum* and their corresponding *ppk*-defective strains under stress conditions was determined as previously described (Alcantara et al., 2014). Cells from single colonies were inoculated in MRS medium and grown at 30°C for 16 h (stationary phase). Subsequently, the cultures were centrifuged, washed twice with one volume of peptone water, suspended in peptone water and inoculated to a final OD_595_ of 0.05 in 250 μl aliquots of growth media dispensed in 96 well microtiter plates. The stress conditions assayed were: growth in MRS supplemented with 0.8 M NaCl, and MRS adjusted to pH 4.0. Growth in MRS medium adjusted to pH 6.5 was taken as a reference. No antibiotics were used in growth assays. No revertants to wild-type were detected under these experimental conditions for the duration of the assay. Growth was monitored by changes in OD_595_ in a microtiter plate reader POLARStar (BMG). At least three independent replicates of each growth curve were obtained.

Maximal growth rates were calculated from best-fit lines for log OD_595_ vs. time using linear regression analysis (GraphPad Prism 4). To determine whether the response of the mutant strains to each stress condition assayed was significantly different to that of the wild-type, pairwise two-way ANOVA analyses were performed taking the growth of the parental strains and mutant strains in the reference condition and each of the stress conditions as treatment condition. These analyses allowed determining whether the measured parameters were significantly different among the wild-type strain and each of the mutants (strain variable) and whether the differences in growth parameters between the wild-type strain and each mutant strain were dependent on the treatment (interaction). We considered that a significant difference was detected if the analysis estimated that both the strain variable and interaction were below a *p*-value of 0.01.

### Polyphosphate Synthesis Analyses

Poly-P granules were stained by the Neisser method (Gurr, 1965). Briefly, bacterial cells grown in MEI plates for 48 h were smeared on microscope slides and let air dry. A freshly prepared solution containing one volume of 0.1% methylene blue dissolved in 5% ethanol plus 5% acetic acid, and two volumes of 0.33% crystal violet in 10% ethanol was added. After 1 min, this solution was rinsed with water and bacteria were stained with a solution of 0.3% chrysoidin G. The slides were washed, dried and observed under a light microscope at ×100 magnification.

Poly-P was isolated from cells grown in MEI medium as previously described (Alcantara et al., 2014) and analyzed in 8% polyacrylamide gels containing 8 M urea in 1 × Tris-borate-EDTA (TBE) buffer, that were stained with toluidine blue (Ghorbel et al., 2006).

### Mercury Sensitivity and Mercury Accumulation

Wild-type lactobacilli strains and their respective *ppk* mutants were inoculated in MEI plates in an agar overlay of 3 ml of MEI medium containing 0.8% agar and 10^6^ CFU. Whatman 3MM paper disks (5 mm diameter) were impregnated with 5 μl of a solution containing Hg(NO_3_)_2_ [Merck, Hg(II)] and CH_3_HgCl (Alfa Aesar, CH_3_Hg) at 1,000 mg/l and deposited on the surface of the agar overlay. The plates were subsequently incubated at 37°C for 48 h. Mercury sensitivity was measured by determining the extension of the growth inhibition halos. Statistical significance determination was performed by means of Student’s *t*-test (GraphPad Prism 4).

Mercury accumulation was measured in bacterial cells grown in MEI medium and suspended in PBS to different final OD_550_ (0.3, 1.25, and 5). After addition of Hg(II) or CH_3_Hg to a final concentration of 1 mg/l, the bacterial suspensions (1 ml) were incubated at 37°C for 1 h, washed with 1 ml of PBS and cells were recovered for quantification. Mercury quantification was performed by cold vapor atomic fluorescence spectrometry (CV-AFS, Millennium Merlin PSA 10.025, PS Analytical, United Kingdom). To this end, samples were digested using a microwave accelerated reaction system (MARS, CEM, Vertex) in teflon reactors in which 4 ml of 14 M HNO_3_ (Merck) and 1 ml of H_2_O_2_ (30% v/v, Prolabo) were added, followed by irradiation at 180°C for 15 min. Samples were made up to volume with 0.6 M HCl. The following analytical conditions were used: reducing agent, 2% (m/v) SnCl_2_ (Scharlab, Scharlau Chemie) in 1.8 M HCl (Merck), 4.5 ml/min; reagent blank, 0.6 M HCl, 9 ml/min; carrier gas, argon, 0.3 l/min; dryer gas, air, 2.5 l/min; delay time, 15 s; analysis time, 40 s; memory wash time, 60 s. Quality control for quantification by CV-AFS was performed by analysing a liquid reference material (QCI-049-1 Trace Metals AA Sample 1, LGC Standards) with a certified mercury concentration of 40.8 ± 1.19 μg/l.

### Quantification of HSP27 Expression

Lactobacilli were grown for 16 h in 10 ml of MEI medium and the supernatants were obtained by centrifugation at 6,000 × *g* 10 min followed by filtration through 0.2 μm pore-size filters. For the *ppk* mutants the inoculum (employed at 1:100 v/v) was grown in MEI containing erythromycin at 2.5 μg/ml, while no antibiotic was added to the final growth medium. Caco-2 cells were seeded at 8 × 10^4^ cells per well in 24-well culture plates with Dulbecco’s Modified Eagle’s medium (DMEM High glucose, Na-pyruvate, Gibco) supplemented with 10% (v/v) fetal bovine serum and 1% (v/v) L-glutamine 200 mM solution (Gibco). Plates were incubated at 37°C in a CO_2_ incubator until they reached confluence (6–7 days) with medium changes every 2 days. Bacterial supernatants (three independent cultures) were neutralized with NaOH solution and diluted 1:10 in DMEM medium. One milliliter aliquots of each diluted replicate were added to the cell cultures and incubation proceeded for 18 h. Cells from the wells were washed with PBS and lysed by adding 50 μl of SDS-PAGE loading buffer per well. Samples were boiled for 5 min and proteins separated in 10% SDS-PAGE gels. After electrophoresis, proteins were transferred to Hybond ECL nylon membranes (GE Healthcare). The blots were incubated with a rabbit polyclonal anti-HSP27 serum (Sigma) at 1:10000 dilution or with a mouse monoclonal anti-β-actin antibody (Sigma) at 1:10000 in TBS supplemented with 0.1% Tween 20 (TTBS) and 5% (w/v) skim milk. After washing with TTBS, secondary antibodies, peroxidase-conjugated anti-rabbit IgG and anti-mouse IgG (GE Healthcare), respectively, were used and the blots were developed with ECL-prime detection reagent (GE Healthcare) with image capturing in a Proxima 2750T apparatus (Isogen Life Sciences). Quantification of protein expression in the blots was performed with Nis Elements BR 3.22 software (Nikon). Statistical significance of differences between wild type strains and their *ppk* mutants was assessed by Student’s *t*-test (GraphPad Prism 4).

### Poly-P Determination in Culture Supernatants

Supernatants of lactobacilli strains grown in MEI medium, obtained by centrifugation and filtration, were subjected to phenol and chloroform:isoamyl alcohol (24:1) extraction and the aqueous phases were transferred to fresh tubes. Polyphosphate was precipitated by adding sodium chloride to 0.1 M final concentration and one volume of ethanol 96% (v/v). The tubes were incubated for 3 h at −20°C and centrifuged for 10 min at 13.000 × *g* at 4°C. The poly-P pellets were washed with 70% cold ethanol and air-dried before being resuspended in 50 μl of water. Poly-P was quantified by fluorescence using 4′,6-diamino-2-fenilindol (DAPI) at a final concentration of 10 μM in 50 mM Tris-HCl pH 7.5, 50 mM NaCl buffer with an excitation wavelength of 415 nm and emission at 550 nm (Aschar-Sobbi et al., 2008) in a Clariostar fluorimeter (BMG LabTech). Serial dilutions of a sample of poly-P isolated from strain Lpp+ were hydrolyzed with a volume of 2 M HCl and incubation at 95°C 15 min, followed by neutralization by adding half volume of 2 M NaOH. The released phosphate was measured with the BIOMOL Green kit (Enzo Life Sciences) as recommended by the manufacturer. This quantified sample of poly-P was used to build a standard curve for the DAPI method.

## Results

### Construction of *Lactobacillus* Mutants Unable to Accumulate Polyphosphate

**FIGURE 1:**
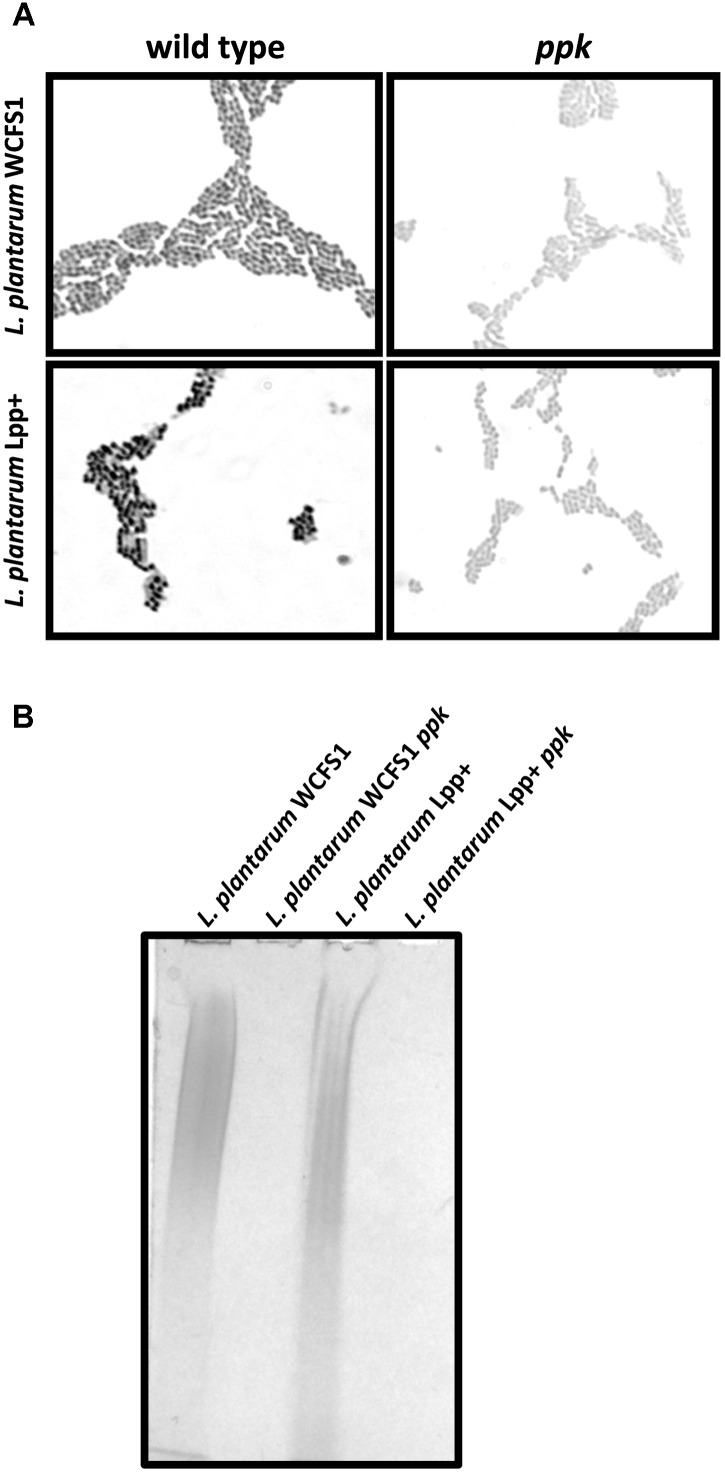
Polyphosphate accumulation in *Lactobacillus.* **(A)** Micrographs (100×) of Neisser-stained *L. plantarum* WCFS1 and *L. plantarum* Lpp+ and their respective *ppk*-disrupted strains grown on MEI plates. The synthesis of poly-P in the wild-type strains is evidenced by the formation of dark cytoplasmic granules revealed by the specific staining. **(B)** Polyacrylamide-urea gel electrophoresis of poly-P isolated from different strain. The gels were stained with toluidine blue.

We had previously obtained a defective mutant in the poly-P kinase (*ppk*) gene in *Lactobacillus casei* BL23 that showed inability to synthesize poly-P and was therefore unable to form poly-P granules in its cytoplasm (Alcantara et al., 2014). Here, we inactivated the *ppk* gene in two additional strains of a different species: *Lactobacillus plantarum*. WCFS1 is an *L. plantarum* strain isolated from human intestine that has been thoroughly characterized at the genetic level and possesses probiotic characteristics (Kleerebezem et al., 2003; van den Nieuwboer et al., 2016). As a second strain we chose an *L. plantarum* strain (Lpp+) isolated in our laboratory which showed a high proportion of poly-P granules positive cells regardless of the culture conditions (data not shown). In these strains, inactivation of *ppk* led to cells where no granules could be detected after specific poly-P staining, compared to the wild type (**Figure 1A**). Furthermore, contrarily to the wild-type strains, no poly-P could be isolated from these two *ppk*-defective derivatives (**Figure 1B**). These results further confirmed the functionality of *ppk* genes in poly-P synthesis and accumulation in this microbial group.

### Effect of High Salt or Low pH on the Growth of *ppk* Mutants

Wild type strains and their corresponding *ppk*-defective strains were grown under different conditions (0.8 M NaCl or pH 4.0) in order to evaluate their ability to grow under stress conditions. The assay was designed so that the growth conditions always had a significant effect on the maximal growth rate. The *ppk* mutation significantly affected the growth under the tested conditions of *L. casei* BL23 (**Table 2**). In contrast, inactivation of *ppk* did not affect the growth of the *L. plantarum* strains (**Table 2**). These results suggest that poly-P accumulation has different physiological effects on *L. casei* and *L. plantarum* that result in a different tolerance to stress conditions.

**Table 2:** Maximal growth rate values and pairwise two way ANOVA at different growth conditions.

Strain	Growth condition						
	MRS	MRS 0.8 M NaCl			MRS pH 4.0		
			ANOVA^1^			ANOVA	
	μ_max_^2^	μ_max_	Strain	Interaction	μ_max_	Strain	Interaction
BL23	0.55 (0.02)^3^	0.32 (0.01)			0.27 (0.02)		
WCFS1	0.74 (0.02)	0.50 (0.01)			0.40 (0.01)		
Lpp+	0.73 (0.01)	0.27 (0.01)			0.24 (0.02)		
BL23 *ppk*	0.69 (0.02)	0.29 (0.01)	<0.001	<0.001	0.24 (0.06)	0.006	<0.001
WCFS1 *ppk*	0.76 (0.02)	0.45 (0.01)	0.629	<0.044	0.40 (0.02)	0.408	0.360
Lpp+ *ppk*	0.69 (0.02)	0.27 (0.01)	0.106	0.184	0.27 (0.01)	0.509	0.039

*^1^p-values of the contribution of the strain and the interaction to the total variance. Contribution of the growth condition is omitted since it was always significant. ^2^Maximal growth rate. ^3^Standard error values are given in parentheses.*

### Mercury Sensitivity in *ppk* Mutants

**FIGURE 2:**
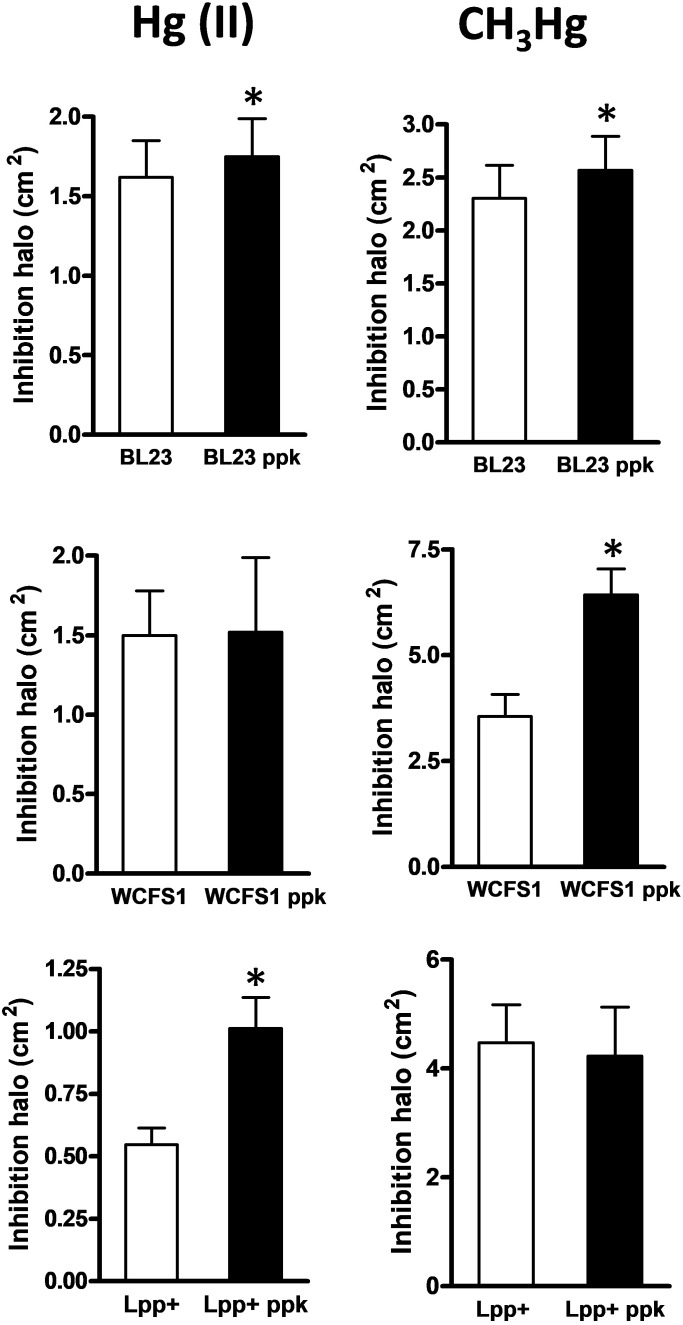
Growth inhibition by mercury in *Lactobacillus* strains and their respective *ppk* mutants. The graphs represent the areas of the inhibition halos produced by inorganic [Hg(II), left column] and organic (CH_3_Hg, right column) mercury in wild type and *ppk* strains. Data are means plus standard deviations (*n* = 20–55). The asterisks indicate a significant difference (*p* < 0.01).

The growth inhibitory effects of inorganic [Hg(II)] and organic [CH_3_Hg] mercury on *L casei* BL23, *L. plantarum* WCFS1, *L. plantarum* Lpp+ and their derivative *ppk*-defective strains was tested in MEI plates, a condition where the three strains produced an abundance of poly-P granules (Alcantara et al., 2014) (**Figure 1**). A significantly increased inhibition of the *ppk* mutants derived from strain BL23 and Lpp+ was observed with Hg(II) (**Figure 2**), indicating a higher toxicity of this metal in the absence of poly-P on these two strains whereas the inactivation of *ppk* in *L. plantarum* WCFS1 did not affect the Hg(II) sensitivity of this strain. In contrast, *L. plantarum* WCFS1 and *L. casei* BL23 *ppk*-defective mutants were significantly more sensitive to CH_3_Hg than the wild-type strain, whereas no differences in sensitivity to CH_3_Hg were observed for the Lpp+ strain (**Figure 2**).

### Mercury Sequestration in *ppk* Mutants

**FIGURE 3:**
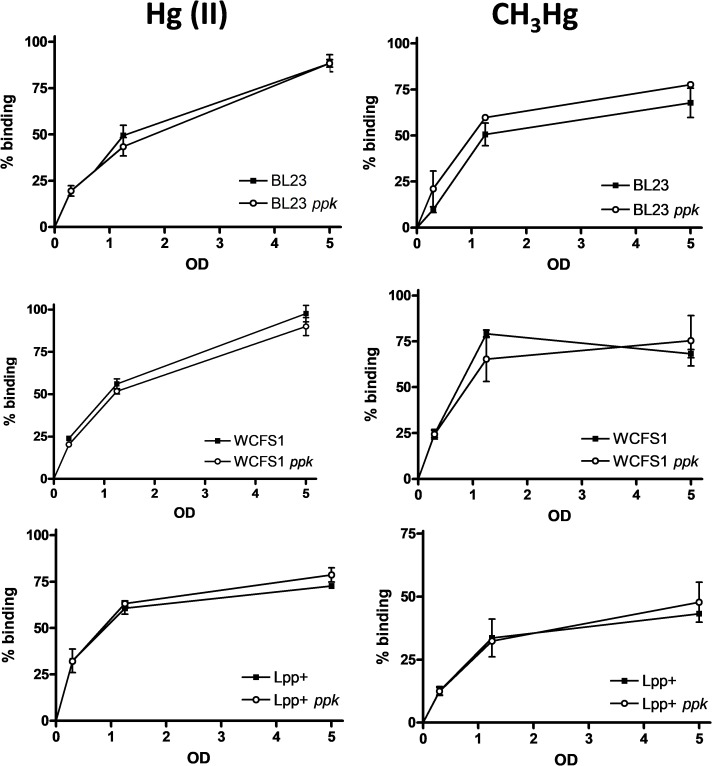
Accumulation of mercury in *Lactobacillus* strains. Lactobacilli were incubated at different optical densities (OD) with different forms of mercury (inorganic, left column and organic, right column) at 1 mg/l and the amount of the metal bound to bacterial cells was quantified. The data represent the percentages of bound metal plus standard deviations from two independent experiments.

We hypothesized that the greater sensitivity to Hg(II) or CH_3_Hg in some of the *ppk* mutants could reflect a diminished capacity of mercury complexation that would result in an enhanced toxicity due to the presence of higher concentrations of free mercury in the cytoplasm. However, the capacity to bind both mercurial forms was not affected by the *ppk* mutation in any tested strain even at low cell densities (**Figure 3**). No differences were observed in the amount of retained mercury forms between wild-type strains and their corresponding *ppk*-defective derivatives, which remained high and dependent on the bacterial densities under our assayed conditions.

### Deficiency in Polyphosphate Synthesis and HSP27 Induction in Caco-2 Cells

**FIGURE 4:**
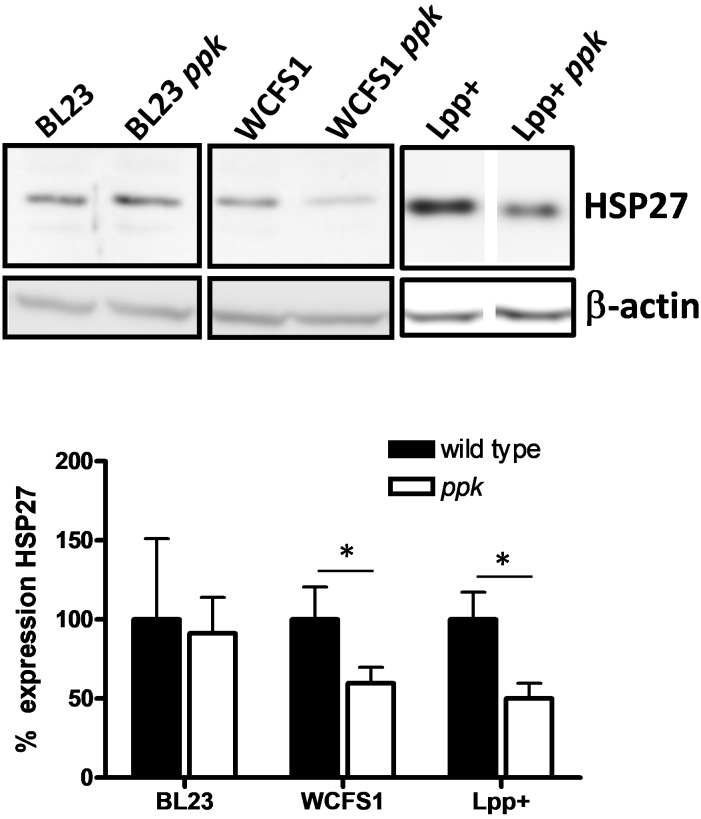
Expression of HSP27 in Caco-2 cells. Monolayers of cultured Caco-2 cells were incubated with supernatants of different *Lactobacillus* and their *ppk*-derivatives and expression of HSP27 was determined by Western blot in crude extracts. Blots developed with anti-β-actin antibody were used as loading controls and they also served to normalize expression. Protein signals in the blots for each pair of strains come from the same gel. The graph below the blots shows the relative HSP27 expression from three replicates (expression in wild-type strains set at 100%). Asterisks indicate a significant difference between wild type and *ppk* strains (*p* < 0.05).

One of the effects observed for *L. brevis*-derived poly-P was the induction of the heat-shock and cytoprotective protein HSP27 in cultured intestinal epithelial cells (Segawa et al., 2011). We tested whether supernatants of wild-type lactobacilli and their mutant strains unable to synthesize poly-P differed in their capabilities to induce this protein in Caco-2 cultures. The addition of conditioned culture supernatants of *L. plantarum* *ppk* mutants derived from strains WCFS1 and Lpp+ resulted in lower levels of HSP27 compared to their parental strains, whereas no differences were observed for *L. casei* BL23 (**Figure 4**). Measurement of poly-P in bacterial culture supernatants showed that *L. casei* BL23 displayed the lowest amount of excreted poly-P (0.12 nmol Pi/ml) compared to *L. plantarum* WCFS1 and Lpp+ strains (0.25 and 0.19 nmol Pi/ml, respectively).

## Discussion

Poly-P synthesis in microorganisms has been widely studied due to their relevant functions in bacterial physiology and as a biotechnological tool in some microbial processes. In this work, we tested functional aspects of probiotic lactobacilli that have been postulated to be linked to poly-P production. Our results reinforced previous research showing that a *ppk* mutation in *L. casei* resulted in a decreased resistance to several stresses (Alcantara et al., 2014) and proposing a link between poly-P and stress resistance in *L. rhamnosus* (Correa Deza et al., 2017). However, this phenomenon does not seem to apply to all species because, contrarily to *L. casei*, no effect on the resistance to NaCl or acidic stresses was evidenced for two *L. plantarum* strains. Furthermore, we established that sensitivity to inorganic and organic mercury was affected by *ppk*, although the extent of the effect varied with the strains and it was metal form-dependent (**Figure 2**). Poly-P synthesis has been proposed to be related to the capacity of some bacteria, fungi and algae to tolerate and sequester heavy metals (Zhang and Majidi, 1994; Aviles et al., 2005; Dodge and Wackett, 2005), although clear evidence on the mechanisms is still lacking in most cases. Increased sensitivity to mercury in *ppk* strains might be explained by different processes. Poly-P metabolism may relieve the direct toxic effects by participating in the mechanisms of stress tolerance to overcome cell damage (e.g., oxidative stress caused by heavy metals; Gray and Jakob, 2015). Alternatively, poly-P may reduce the intracellular effective concentration of mercury through complexation. In *E. coli*, Cu^2+^ tolerance depends on poly-P. It has been demonstrated that Cu^2+^ chelation by poly-P reduces copper toxicity, and that the metal is expelled from the cells as Cu^2+^/PO_4_^3−^ complexes in a reaction catalyzed by the PitAB phosphate transporter after hydrolysis of poly-P by the exopolyphosphatase Ppx (Grillo-Puertas et al., 2014). Our results, however, suggest the poly-P effect on protection against mercury damage may not be direct or it may require the participation of additional factors. First, the effect of *ppk* inactivation on mercury toxicity varied in each strain used in this study (**Figure 2**). These differences are difficult to explain if poly-P has a direct role in protection. Second, no significant difference in mercury accumulation was observed between the wild-type strains and their respective *ppk*-defective mutants (**Figure 3**).

Our observations indicate that the poly-P-influenced mechanisms that affect mercury resistance would not necessarily ave an impact on the capacity to accumulate mercury. It has been recently shown that lactobacilli can efficiently sequester Hg(II) as well as CH_3_Hg, the main mercurial form found as food contaminant (Alcantara et al., 2017; Jadan-Piedra et al., 2017). However, our results question the role of poly-P in the mercury retention mechanism. With the exception of the increased mercury accumulation in recombinant *E. coli* overexpressing *ppk* (Pan-Hou et al., 2002; Ruiz et al., 2011), direct proofs of poly-P/Hg complexation in microorganisms are lacking. It has been postulated that binding of heavy metals by lactic acid bacteria occurs mainly via surface interactions with cell wall components and that low proportions of the toxic are internalized through processes that may involve specific/unspecific transport or diffusion (for review, see: Mrvcic et al., 2012; Zoghi et al., 2014; Chiocchetti et al., 2018). This notion has been specifically proven for *L. casei* BL23 (Alcantara et al., 2017). Since most poly-P is accumulated intracellularly as cytoplasmic granules, it would therefore have a low interaction with mercury. Furthermore, analysis of poly-P accumulation in *Lactobacillus paracasei* JCM1163 showed that the amount of poly-P in culture supernatants was 1,000-fold lower than that found in the cytoplasm (Saiki et al., 2016).

Research on the probiotic mechanisms of action has led to the discovery of a number of molecules implicated in the cross-talk between bacteria and host cells (Bron et al., 2011; Lebeer et al., 2018). Poly-P has been identified as one of these soluble probiotic factors. Poly-P produced by *L. brevis* induced HSP27 and increased the intestinal barrier function in Caco2/bbe cells (Segawa et al., 2011; Tanaka et al., 2015). Furthermore, it has been shown that poly-P diminished intestinal inflammation in models of chronic colitis (Kashima et al., 2015), and that inhibited tumoral SW620 cells viability (Sakatani et al., 2016). By means of the use of other *Lactobacillus* species (*L. plantarum*) and their respective *ppk* mutants, we showed that supernatants of strains devoid of poly-P displayed a diminished induction of HSP27 in cultured intestinal cells compared to the wild type. This supports the notion that poly-P is a probiotic factor shared by other poly-P producing *Lactobacillus* species in addition to *L. brevis*. Differences in the effectiveness between strains (i.e., *L. casei* BL23) are possibly due to the different amounts of poly-P that they can produce and secrete. Therefore, it would be interesting to search for isolates with high capacity of poly-P synthesis. In this regard, the isolation of *L. paracasei* JCM1163 mutants which showed an increased capacity to synthesize poly-P has been reported although they have not been characterized at the genetic level (Saiki et al., 2016).

Although we have not evidenced a role for poly-P in heavy metal sequestration, lactobacilli-derived poly-P emerges as a factor mediating mechanisms of stress tolerance and as a likely probiotic factor in species possessing *ppk* genes. Owing to the relevance of lactobacilli in food and health, knowledge of the many poly-P functions in this microbial group will help to understand their physiology and interactions in bacterial communities and symbiotic relationships. These would cover positive but also negative aspects, as it has been recently described that poly-P accumulation in dental biofilm bacteria such as *Lactobacillus rhamnosus* may participate in teeth demineralization during carious lesion progression (Breiland et al., 2018).

## Author Contributions

VM, MZ, VD, and DV designed the work. CA, JC-M, CJ-P, and VM performed the experiments. VM and MZ wrote the manuscript.

## Conflict of Interest Statement

The authors declare that the research was conducted in the absence of any commercial or financial relationships that could be construed as a potential conflict of interest.
